# Characterization of GaAs Solar Cells under Supercontinuum Long-Time Illumination

**DOI:** 10.3390/ma14020461

**Published:** 2021-01-19

**Authors:** Nikola Papež, Rashid Dallaev, Pavel Kaspar, Dinara Sobola, Pavel Škarvada, Ştefan Ţălu, Shikhgasan Ramazanov, Alois Nebojsa

**Affiliations:** 1 Department of Physics, Faculty of Electrical Engineering and Communication, Brno University of Technology, Technická 2848/8, 61600 Brno, Czech Republic; nikola.papez@vutbr.cz (N.P.); xdalla03@stud.feec.vutbr.cz (R.D.); kasparp@feec.vutbr.cz (P.K.); sobola@feec.vutbr.cz (D.S.); skarvada@feec.vutbr.cz (P.Š.); 2 CEITEC BUT – Brno University of Technology, Purkyňova 656/123, 61200 Brno, Czech Republic; alois.nebojsa@ceitec.vutbr.cz; 3 Department of Inorganic Chemistry and Chemical Ecology, Dagestan State University, Makhachkala, St. M. Gadjieva 43-a, 367015 Dagestan Republic, Russia; ramazanv@mail.ru; 4 Directorate of Research, Development and Innovation Management (DMCDI), Technical University of Cluj-Napoca, Constantin Daicoviciu Street, no. 15, Cluj-Napoca, 400020 Cluj County, Romania; 5 Department of Solid State Physics and Surfaces, Faculty of Mechanical Engineering, Brno University of Technology, Technická 2896/2, 61600 Brno, Czech Republic

**Keywords:** gallium arsenide, illumination, supercontinuum, electrical characteristics, xps, Raman spectroscopy, sims

## Abstract

This work is dedicated to the description of the degradation of GaAs solar cells under continuous laser irradiation. Constant and strong exposure of the solar cell was performed over two months. Time-dependent electrical characteristics are presented. The structure of the solar cells was studied at the first and last stages of degradation test. The data from Raman spectroscopy, reflectometry, and secondary ion mass spectrometry confirm displacement of titanium and aluminum atoms. X-ray photoelectron spectroscopy showed a slight redistribution of oxygen bonds in the anti-corrosion coating.

## 1. Introduction

The effects of radiation exposure of optical elements, such as GaAs solar cells, represent an essential parameter for the evaluation of stability and efficiency. We have already reported the degradation of GaAs solar cells under temperature and gamma radiation [1,2,3]. Although GaAs photovoltaic cells are smaller in the field of solar cells, it can be argued that they dominate in terms of special applications such as stability and resistance to various effects of electromagnetic radiation. Examples include satellites, rovers, and other space devices [4]; military; aerospace; and high-concentration photovoltaic (HCPV) systems [5]. In most of these cases, solar cells are exposed to extreme radiation and other stresses. These are also sectors in which very strict operating conditions are imposed. Continued study of the stability of cells of this type and investigation of other effects of degradation is, therefore, more than desirable. Likewise, it was proven that the surface and thin anti-reflective layers of a single-junction GaAs solar cell are changing during highly ionizing electromagnetic radiation [6]. This study includes a description of GaAs solar cells aging under irradiation by supercontinuum laser (SL). The SL becomes a part of solar simulator when traditional lamps or light-emitting diodes are not suitable due to low spatial coherence of radiation [7].

The SLs combine the efficiency, power, and coherence of lasers with broad-spectrum coverage of conventional broadband light sources while being able to exceed their brightness by several orders of magnitude without losing the option of being used for delicate optical microscopy applications. Currently, it is a unique technology, providing high power and the broad spectrum of light with very short pulse duration. They are used for a wide variety of purposes, especially in medical and biological fields. Applications of SL include, but are not limited to, fluorescence lifetime imaging and confocal microscopy, optical coherence tomography, measurements within plasmonics and metamaterials, carbon nanotubes, quantum dots, and others. The properties of SL are a huge asset in many fields, using its coherent white light as a source for optical coherence tomography [8], optical communication [9], or a probe for excitation in chemistry and biology [10], to name a few.

Absorption of light radiation by solar cells can be accompanied by heating. Recently, the degradation of GaAs solar cells under continuous laser irradiation was discussed by Lei Qi et al. [11] in terms of thermal-stress distributions. Here, we discussed the degradation caused by defects formed due to redistribution of aluminum and titanium components. Light-induced degradation of silicon solar cells was reported to be connected with increasing of dislocation defects [12]. Electron traps that appear due to irradiation can relax over time, and the bond between electrons and the lattice of the material becomes weaker. The traps originate from the displacement of an atom when the kinetic energy of radiation is sufficient, and Frankel pairs can appear [13].

## 2. Results and Discussion

**Figure 1:**
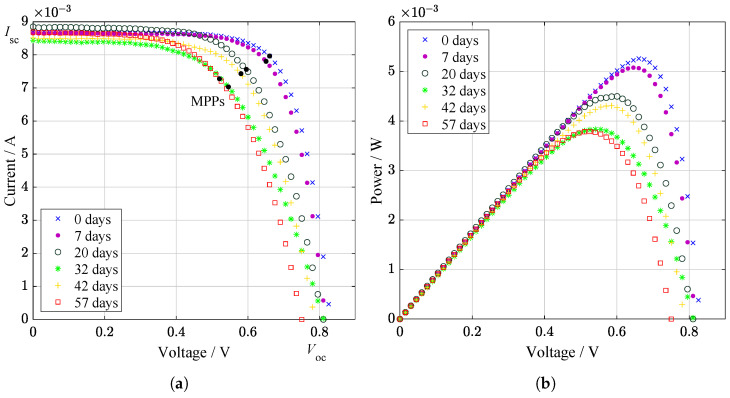
Light I–V curves from supercontinuum laser irradiation. In subfigure (**a**), I–V characteristics are indicated as maximum power points (MPPs). In subfigure (**b**), the power characteristics show a slight increase in performance may be observed during 42th day.

**Figure 2:**
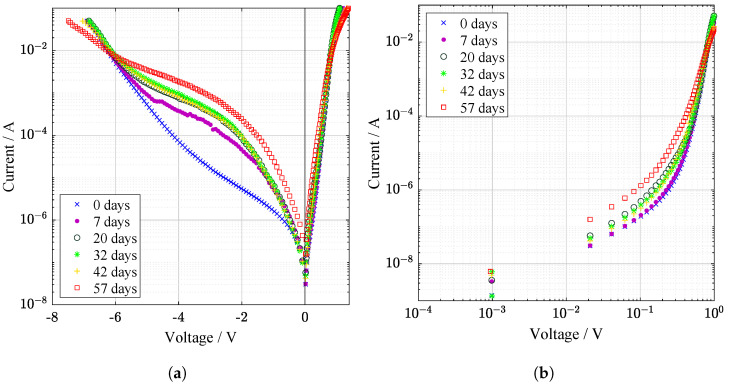
Dark I–V curves from supercontinuum laser irradiation in (**a**) semi-logarithmic and (**b**) loglog scale. Slight relaxation and improvement during 20 to 42th day is observed.

The current–voltage and power characteristics were studied under illumination (Figure 1). The difference in the internal resistance of the samples caused by the redistribution of titanium and aluminum atoms affects the performance. The minor carriers are responsible for the electrical behavior of solar cell under illumination. Generation of electron–hole pair can be affected by defects of the material structure. Defects at the depletion region caused increasing of recombination current and indicates degradation of pn junction (Figure 2). The decreasing in the concentration of charge carriers can be associated with the capture of charge carriers on the resulting defects. Low effective lifetime of carriers can also be connected with defects caused by the migration of Al and Ti atoms. Electrical characteristics demonstrate a nonlinear character of degradation [14]. It was most probably due to the fact that including of Al caused the appearance of deep donor level centers (DX centres). They play an essential role and, as was shown by J. Y. Lin et al. [15], are responsible for persistent photoconductivity in amorphous Al${}_{x}$Ga${}_{1-x}$As. The hybridizations for two-site atoms produce changes in charge state and bonding that explain a wide range of effects. Diffusion of titanium creates additional charge separation in the film. The phase transformation of the Ti−O superlattice from the anatase phase to rutile may occur at the interface, which contributes to a change in the efficiency [16]. In this case, Ti atoms are partially released from the structure, which is confirmed by secondary ion mass spectroscopy (SIMS) [17,18].

### 2.1. Electrical Characteristics

The power drop before and after irradiation can be seen from the light illuminated electrical characteristics. Light curves are divided into I–V characteristics in Figure 1a and P–V characteristics in Figure 1b. Important parameters related to light electrical characteristics are listed in Table 1. A similar pattern of decline was measured for dark characteristics in semi-log (Figure 2a) and log-log (Figure 2b) plot. There are no light fluctuations, like in illuminated measurements, where is a significant amount of noise. This is a more accurate method, where no solar simulator is used but the injection of carriers. It is a method for mainly determining the quality of junction and contact resistivity.

We reported light-induced degradation which occurs when a solar cell is for the first time exposed to solar radiation. Study of stability of GaAs-based structures during a short period (1–2 days) was recently investigated by Lang et al. [19]. This is first stage of the panel “tune-up” or “initial degradation”. In our test case the stabilization period was 20–32 days. This is typical for monocrystalline solar cells, silicon hydrogenated solar structure, as well as for GaAs-based solar cells. According to review of Hussin et al. [20], the stabilization can take anywhere from weeks up to few months from the beginning of the solar cells exploitation. For these reasons, we were waiting for the remarkable changes, which came, as expected, after the stabilization period. Then, we were able to describe degradation using structure characterization methods. It worth to note, that even these slight changes can influence the module performance at large scale solar panels.

From Figure 2a, decay after stabilization period is caused by chemical processes in the heterojunction, whereas the time of stabilization can be associated with metastable effects caused by light-induced degradation.

### 2.2. Raman Spectroscopy

**Figure 3:**
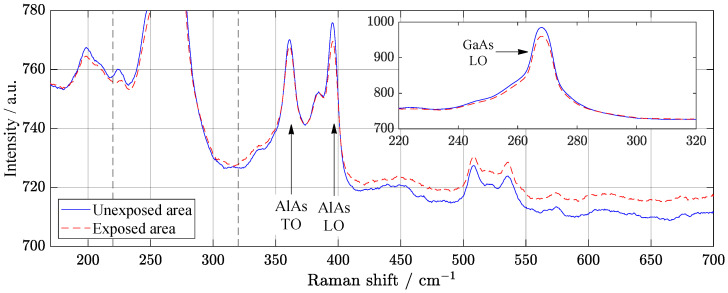
Raman spectroscopy from specimen processed by supercontinuum laser irradiation. Due to the height of the transverse-optical mode of GaAs, in order to preserve detail, the peak was plotted in a separate graph. Differences in relative intensities of AlAs phonons indicates possible displacements at As sites. Exposed area is measured after SL illumination of 67 days.

Raman spectroscopy was performed to control the presence of structural defects and free charge carriers. Considering the relative intensity of localized vibrational modes provides information about defects of nature. Scattering at longitudinal optical phonons and changes in layer thickness as a result of aluminum diffusion can cause energy losses. The decrease in these phonons’ intensity, as shown in Raman spectra, may explain the temporary improvement in the performance of solar cell under illumination (Figure 1). Change of local vibrational modes in Raman spectra can also be associated with DX centers. The transverse optical (TO) phonon of GaAs stays similar asymmetric form before and after SL irradiation (Figure 3). It is possible to observe the small difference in relative intensities of AlAs phonons indicates that displacements may occur at As sites. Low-intensity broad peaks around 500 to 580 cm${}^{-1}$ belong to second-order GaAs-like TO and longitudinal optical (LO) phonons [21].

Here, we consider the degradation of the surface and near-surface area of the solar cells under illumination. Surface recombination influences loss processes in GaAs solar cell as well as other factors (thermalization, Joule heat, bulk recombination, and Peltier heat) [22]. Raman spectroscopy is a powerful tool for studying structural variations of GaAs structures [23,24]. Espinosa-Vega et al. [25] noted that small changes of Raman modes indicate the perfection of GaAs structures. The ratio of phonons could be an indicator of GaAs structural composition [26].

### 2.3. Spectrophotometry

**Figure 4:**
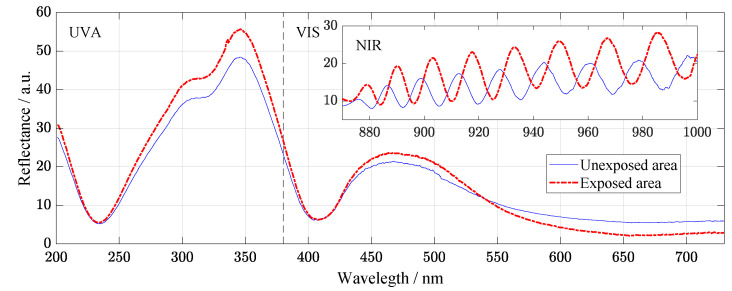
Reflectance measurement from supercontinuum laser irradiation. There is a significant change, especially in the near-infrared (NIR) region. These interference fringes indicate a differences in thin-films. Exposed area is measured after SL illumination of 67 days.

The spectrophotometric measurement shown in Figure 4 was performed on a wide spectral range from 200 to 1000 nm. The reflectivity differences between the irradiated and non-irradiated areas from the ultraviolet (UVA) to the visible spectrum (VIS) can be considered negligible. Still, a much more interesting area is marked in the near-infrared (NIR) light range. Number and amplitudes of the fringes slightly differ, and a small shift to higher wavelength is observed. Larger intensity is related to the degradation of anti-reflective coating of the samples. The number of fringes increased due to changing of the layers interface areas and their thickness, and, at the same time, the shift of the spectra confirms it as the result of damages induced by radiation is consist in displacement and interdiffusion of Ti and Al atoms. The results of the reflectometry are in correlation with element profile studied by SIMS.

### 2.4. X-ray Photoelectron Spectroscopy (XPS)

**Figure 5:**
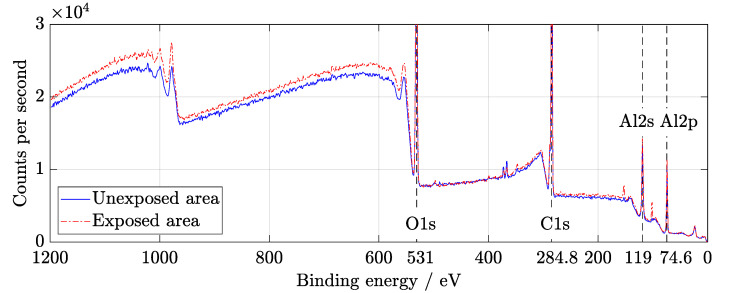
Wide X-ray Photoelectron Spectroscopy (XPS) spectra of exposed and unexposed area from supercontinuum laser irradiation. Four significant peaks are marked: O1s, C1s, Al2p, and Al2s. These regions were then examined in high resolution. Al2p and Al2s peaks are associated with thin anti-reflection layers.

**Figure 6:**
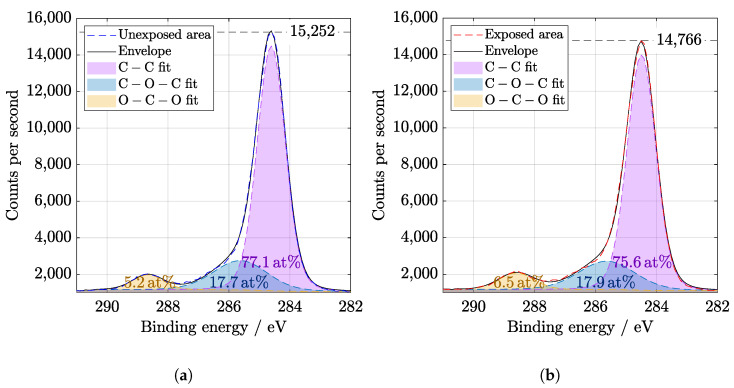
High resolution of C1s region from XPS (**a**) before and (**b**) after SL irradiation of 67 days. Binding energy value for C − C is 284.6 eV, for C − O − C is 285.6 eV, and for O − C − O is 288.6 eV. An increase of last two mentioned bonds is visible.

**Figure 7:**
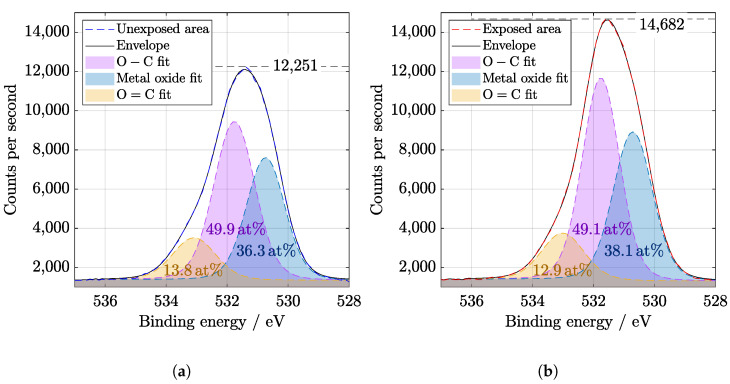
High resolution of O1s region from XPS (**a**) before and (**b**) after SL irradiation of 67 days. Binding energy value for O − C is 531.7 eV, for metal oxide is 530.7 eV, and for O = C is 533 eV. It can be seen there is an increase in the exposed area, compared to the C1s region, where is a slight decrease.

It is known that ${\mathrm{AlO}}_{x}$ is used for passivation of solar cell surfaces [12] due to the excellent protective and anti-corrosive properties of aluminum oxide [27]. XPS wide spectra show the presence of aluminum and oxygen peaks that belong to the coating (Figure 5). Before evaluation, the spectra were calibrated to C1s peak at 284.8 eV. A slight increase in C−O−C and O−C−O bonds could be observed at C1s peak in Figure 6. The components of O1s binding energy (Figure 7) are associated with bonding with carbon and aluminum and in agreement with Al2p, Al2s, and C1s peaks fitting.

**Figure 8:**
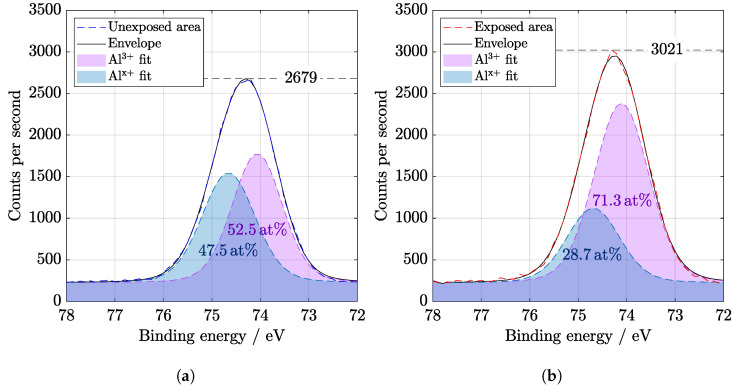
High resolution of Al2p region from XPS (**a**) before and (**b**) after SL irradiation of 67 days. Binding energy value for Al^3+^ is 74.1 eV and for Al^x+^ is 74.6 eV. Displacement of the Al peak indicates the degradation of the thin layer.

**Figure 9:**
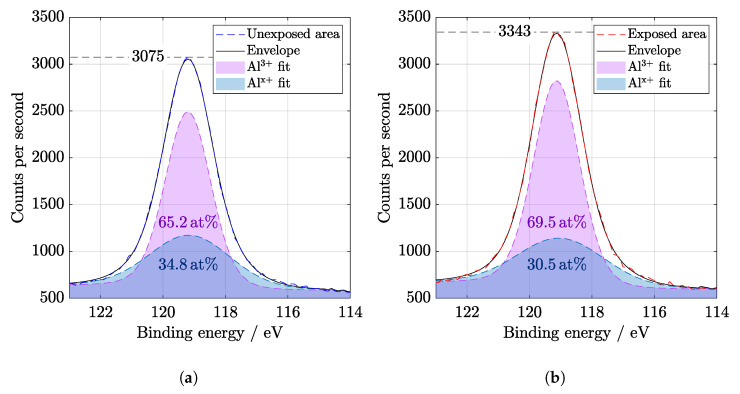
High resolution of Al2s region from XPS (**a**) before and (**b**) after SL irradiation of 67 days. Binding energy value of deconvoluted oxidation states for Al^3+^ is 119.1 eV and for Al^x+^ is 119 eV. The relative change in energy is indicated by a difference in the structure composition of anti-reflection coating elements.

Both Al2p and Al2s peaks in Figure 8 and Figure 9 were deconvoluted to ${\mathrm{Al}}^{3+}$ and ${\mathrm{Al}}^{x+}$ oxidation states. Relevant results were also provided, for example, by Dallaeva et al. [27], where structural properties of ${\mathrm{Al}}_{2}O_{3}$ were also studied. The amount of aluminum suboxides bonds [28] is lower after illumination. Nevertheless, a slight displacement of the Al peak indicates the degradation of the film. A change of the binding energy indicates a relative loosening of the structure because the elements from the anti-reflection coating diffused into the depth.

### 2.5. Secondary Ion Mass Spectroscopy (SIMS)

**Figure 10:**
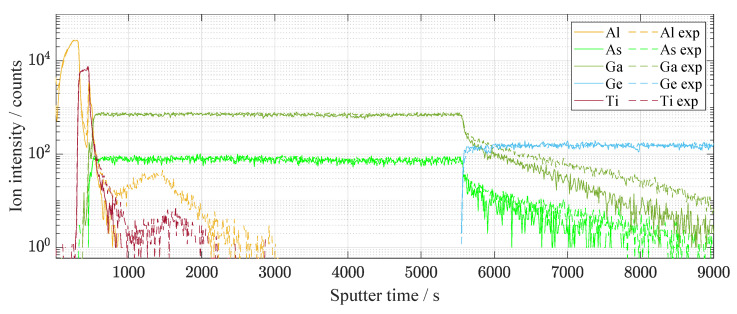
The whole etching process using SIMS in time dependence. The graph is in semi-logarithmic representation. The etched was exposed (illumination of 67 days, dashed line) and unexposed area. The graph is represented for a basic idea of the etching process where the etching depth was up to 8 μm. On the contrary, the second graph in Figure 11 is used to recognize the main differences in thin-films between the exposed and unexposed areas.

**Figure 11:**
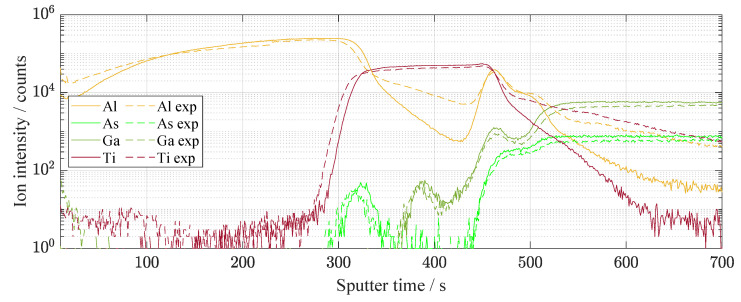
The first few etched tens of nanometers of the solar cell surface in detail using SIMS. The change in Al is especially visible. The difference is visible before and after illumination of 67 days.

Light exposure produces the defects caused by the displacement of atoms. It influences a charge distribution at the interfaces of the pn junction. The SIMS analysis was carried out several times in order to avoid the effect of local defects on the results [29]. Figure 10 shows the distribution of elements along with all solar cell from the top thin-layers to the substrate in the GaAs/Ge interface. Nevertheless, the most significant changes that influence electrical behavior belong to the thin surface layers. The detailed spectrum is shown in Figure 11. The character of Al and Ti diffusion is mostly anisotropic and tends to AlGaAs layer, changing the concentration of aluminum creating displacement defects. Kinetics of Ti-sinking to GaAs compounds was intensively studied [30,31] before.

## 3. Material and Methods

**Figure 12:**
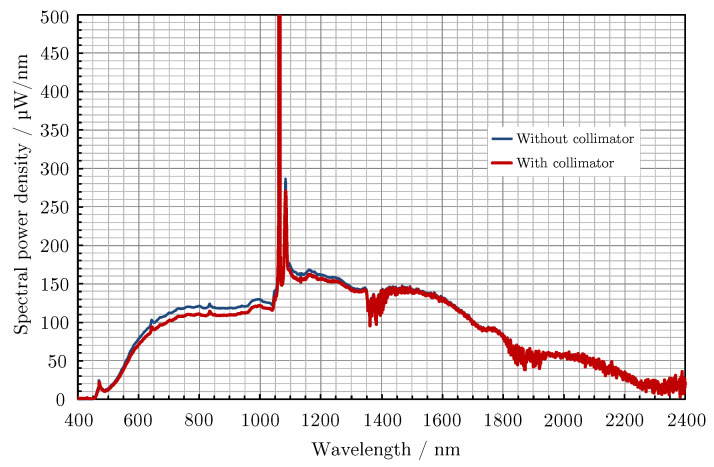
Declared power spectral density from manufacturer of the SL.

The supercontinuum laser used for experiments described in this paper is Leukos Samba 450 optical system with a measured spectral range of 450 nm to 2400 nm and the total average power output 188 mW without collimator. For a basic idea of power at a given wavelength power spectral density from manufacturer is illustrated in Figure 12. The system is capable of operating in single mode and multimode, the latter being needed for full-scale spectrum coverage.

**Figure 13:**
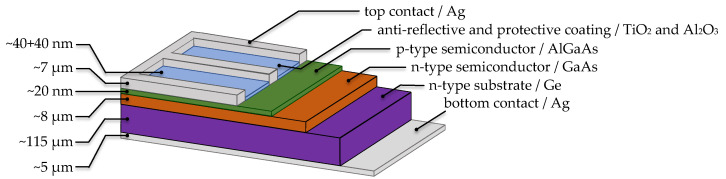
Simplified model structure of the single-junction GaAs solar cell. Essential layers are GaAs/AlGaAs, which create a pn junction, where AlGaAs is only thin layer of 20 nm. An important function of protection and anti-reflection against the adverse effects performs ${\mathrm{Al}}_{2}O_{3}$ and ${\mathrm{TiO}}_{2}$ thin-films.

The samples are commercially available single-junction GaAs-on-Ge with n-type Ge substrate, where the pn junction is created between AlGaAs and GaAs layers as Figure 13 demonstrates. Thin layer of ${\mathrm{Al}}_{2}O_{3}$ and ${\mathrm{TiO}}_{2}$ about 40 nm each serves as anti-reflection coatings (ARC) as well as protection against radiation from space. Due to the single-junction structure, which is excellent for a clear demonstration of the results, we have been studying this special type of solar cell used in satellite applications for several years. The samples were exposed to radiation during 67 days at a distance of 67 mm. The maximum laser power was set to 188 mW. Spot size is 5.73 mm${}^{2}$ at the 200 mm${}^{2}$ square samples. Several measurements were performed in succession on different areas of the solar cell to confirm the results and eliminate measurement errors. No cover glass to the solar cells and no bias were applied during irradiation.

Electrical characteristics were taken for five exposure sessions in order to study changes in performance—before the irradiation, and after 7, 20, 32, 42, and 57 days (Please note that the whole experiment with SL took 67 days, but electrical measurement took 57 days.). Current–voltage (I–V) characteristics were measured under dark and illuminated conditions. All dark and light electrical characteristics were measured at temperature of 25 ${}^{\circ }C$. During the measurement of I–V characteristics under illumination, a standardized light source calibrated to an output of 1000 W/m^2^ was used to simulate sunlight activity. From the characteristics of illumination was also calculated P–V characteristic with the maximum power point. As a measurement system unit was used NI PXIe-1073 with the PXI-4130 SourceMeter and the PXI-6224 data acquisition module. This module can measure voltage, current, temperature, and other electrical or physical parameters depending on the peripherals connected. The dark I–V measurement setup consisted of a shaded and darkened box with a Keithley 2510-AT AUTOTUNING TEC SourceMeter with an automatically controlled temperature. The Peltier plate with a water-cooled radiator was controlled and maintained by a temperature system. For measuring the characteristics the computer-connected Keithley 2420 power supply was used.

Raman spectroscopy was measured by WITec confocal Raman imaging system alpha300 R (WITec, Ulm, Germany) using objective 100×, 532 nm laser with 5 mW power before the objective. Exposure time was 7 s and the number of accumulations 20.

Influence of the irradiation to optical properties was studied by reflectometry using UV–VIS Optical Spectrometer Ocean optics JAZ 3-channel. This non-destructive contactless method provides rapid evaluation of properties, including doping of the materials. A thin gold-coated wafer was used as the calibration sample. Reflectance spectra were measured in the range from 200 nm to 1000 nm. The spectrum of ultraviolet light in the range of 200 nm to 380 nm, visible light in the range of 380 nm to 740 nm, and near-infrared light in the range of 740 nm to 1000 nm were measured.

To study the distribution of the elements at the surface, the AXIS SupraTM X-ray photoelectron spectrometer (Kratos Analytical Ltd., Manchester, UK) was used. XPS spectra were fitted by CasaXPS v.2.3.23 software and all plots processed by Matlab 2020b desktop environment [32]. XPS source used an emission current of 15 mA, investigated spectra between 1200 eV to 0 eV with step of 1 eV. The wide spectrum was always measured on each sample as the first part to give an idea of the complete elemental composition on the sample surface. From the resulting wide spectrum, the regions that were most interesting and important for the given sample within its processing were then identified by elemental binding energy. The spectra has been swept several times for higher accuracy (several identical consecutive measurements).

TOF.SIMS5 set-up (ION-TOF, Muenster, Germany) allows determining changes in the distribution of the elements. Main investigated peaks are ${\mathrm{Ga}}^{+}$, ${\mathrm{Ge}}^{+}$, ${\mathrm{Al}}^{+}$, ${\mathrm{Ti}}^{+}$, and ${\mathrm{As}}^{+}$. These elements appeared mainly in the form of positives ions, so the measurement was done in positive mode. As a primary beam, a ions of ${\mathrm{Bi}}_{1}^{+}$ with the energy of 60 keV was chosen. As a sputter beam, a reactive species of Cs with the energy of 2 keV and 100×100 μm crater size were used.

## 4. Conclusions

The results of two-month irradiation by the continuous laser of single-junction GaAs solar cells were studied. As this is a special type of solar cell used under challenging environments, its analysis is desirable. The sample was examined using several measuring methods, which could complement and confirm each other. The SL is a promising tool for characterization of the solar cells degradation processes. The SL energy caused displacement defects due to migration of Ti and Al atoms. A good agreement between functional (electrical), optical, and structural properties is observed. Summarized dependences of electrical properties on exposure duration show a slight increase in efficiency at 42nd day of the experiment. The XPS spectra show the degradation of the protective ${\mathrm{AlO}}_{x}$ layer as well. Raman spectroscopy allows us to suggest that As sites are related to defects formation. Diffusion of Al and Ti caused the changing of interference fringes studied by reflectometry. Similar results from spectrophotometric measurements were published by Kaur, Mitra, and Yadav where the fringe pattern is permuted when the thickness of the thin-film is changed [33]. The fact of element displacement, as well as the anisotropic character, was shown and confirmed by SIMS. These are the results from one of a set of several years of researches [3,34] of solar cells of the same type, where it can be confirmed that a similar phenomenon was also observed after strong irradiation of gamma rays.

## Figures and Tables

**Table 1:** The essential measured parameters during the illumination process of I–V curves. Each row in the table indicates the relevant day after illumination. The first row where the value is 0 days shows the measurement before the start of the process. Selected parameters are open-circuit voltage $V_{\mathrm{oc}}$, short-circuit current $I_{\mathrm{sc}}$, voltage at MPP $V_{\mathrm{mpp}}$, current at MPP $I_{\mathrm{mpp}}$, power at MPP $P_{\mathrm{mpp}}$, and fill factor FF. The most decreased photovoltaic parameter of the cell can be considered fill factor during the 32nd day. There is a decay after stabilization, which is caused by chemical processes in the heterojunction.

Days	$V_{\mathrm{oc}}$ / mV	$I_{\mathrm{sc}}$ / mA	$V_{\mathrm{mpp}}$ / mV	$I_{\mathrm{mpp}}$ / mA	$P_{\mathrm{mpp}}$ / mW	*FF* / –
0	832.4	8.701	660.6	8.965	5.262	0.727
7	817.5	8.661	650.7	7.803	5.078	0.717
20	810.7	8.851	595.6	7.560	4.503	0.628
32	813.9	8.440	545.5	7.031	3.836	0.558
42	787.7	8.513	580.5	7.432	4.315	0.643
57	750.5	8.687	520.5	7.277	3.787	0.581

## Data Availability

On demand.

## Sample Availability

Samples of the GaAs-based solar cell are available on demand from Nikola Papež. E-mail: Nikola.Papez@vutbr.cz.

## Abbreviations

The following abbreviations are used in this manuscript:

ARC	Anti-reflection coatings
HCPV	High-concentration photovoltaic
LO	Longitudinal optical
MPP	Maximum power point
NIR	Near-infrared
SIMS	Secondary ion mass spectroscopy
SL	Supercontinuum laser
TO	Transverse optical
UVA	Ultraviolet A
VIS	Visible
XPS	X-ray photoelectron spectroscopy
